# Effects of acupuncture on mental health of migraine patients: a systematic review and meta-analysis

**DOI:** 10.1186/s12906-023-04103-8

**Published:** 2023-08-04

**Authors:** Zhao Li, Jie Feng, Shao Yin, Xin Chen, Qicheng Yang, Xu Gao, Deya Che, Li Zhou, Hui Yan, Yue Zhong, Fengya Zhu

**Affiliations:** 1 Hospital of Chengdu University of Traditional Chinese Medicine, Chengdu, China; 2 Acupuncture and Tuina School, Chengdu University of Traditional Chinese Medicine, Chengdu, China; 3 Department of Medical Information Engineering, Chengdu University of Traditional Chinese Medicine, Chengdu, China; 4 Zigong First People’s Hospital, Zigong, China

**Keywords:** Migraine, Acupuncture, Quality of life, Mental health, Meta analysis

## Abstract

**Background:**

Migraine is a neurological disease characterized by moderate to severe headache and various neurological symptoms. It is often cause mood and anxiety disorders that can seriously affect quality of life. Acupuncture has been claimed to have a role in treating neuropsychiatric disorders and is becoming increasingly popular. However, it remains unclear whether current evidence is sufficient to support acupuncture in improving mental health in migraine patients.

**Objectives:**

This systematic review and meta-analysis aimed to investigate the effect of acupuncture on the management of pain and mood disorders in patients with migraine.

**Methods:**

We searched PubMed, Cochrane Library, Embase, Web of Science, Chinese National Knowledge Infrastructure (CNKI) and Wan Fang Data Knowledge Service Platform for reports, conferences and academic papers published before January 1, 2022. Randomized controlled trials (RCTs) including acupuncture, sham acupuncture and medication for migraine were included. Stata 16.0 software and Cochrane RoB2.0 were used for data processing and migration risk analysis.

**Result:**

Thirteen randomized controlled trials containing 1766 migraine patients were included in the present study, the results showed that compared with sham acupuncture and medication, acupuncture seemed to have advantage in improving SAS (WMD: -5.64;95% CI: -10.89, -0.39; *p* = 0.035) and SDS (WMD: -4.65; 95% CI: -9.25, -0.05; *p* = 0.048) in migraine patients. And it seems to be more effective in improving MH (SMD: 0.77; 95% CI: 0.19, 1.35; *p* = 0.009), VAS (SMD: -1.06; 95% CI: -1.73, -0.4; *p* = 0.002;) and MSQ (WMD: 4.76; 95% CI: 2.36, 7.15; *p* < 0.001) than sham acupuncture and medication.

**Conclusion:**

The present results suggest that, compared with Western medicine and sham acupuncture, acupuncture seems to be able to effectively improve anxiety and depression in migraine patients.And it may be more effective in improving SF36-mental health, VAS and MSQ than shame acupuncture or Western medicine. The results of this study need to be verified by higher quality RCTs.

**Supplementary Information:**

The online version contains supplementary material available at 10.1186/s12906-023-04103-8.

## Introduction

Globally, migraine is a common disease, with about 1.3 billion people suffering from them; female patients are 3–4 times more common than male patients [1]. It is the second-highest specific cause of disability worldwide [2]. Depression, anxiety and insomnia often occur in migraine sufferers, especially in those who have chronic migraine [3, 4]. In general, pain is positively correlated with accompanying symptoms. Migraine poses a significant psychological, healthy and financial burden to sufferers. There are many factors that can trigger migraines, with common triggers including stress, disrupted sleep, emotional agitation, menstruation, noise, light, hot and cold weather and fatigue [5–7].

Anxiety is widely believed to increase the burden of migraines. A large prospective study with more than 5000 participants found that anxiety intensity was associated with headaches in individuals with tension-type headaches, migraine and coexisting tension-type headaches and migraine [8]. Migraine predicts depression, while depression also predicts migraine in subjects, a link that is partly mediated by anxiety [9]. In fact, mood disorders and migraines can interact and induce bipolar disorder (BD) in severe cases. Evidence suggests that depression often precedes migraines [10], while previous studies have shown that migraine sufferers have a high incidence of BD; a family history of BD is a risk factor for migraine deterioration [11]. In addition, insomnia is the most common sleep disorder among patients with migraine, in a relationship that appears bidirectional [12]. Evidence supports the suggestion that migraine sufferers have worse sleep quality than non-migraine sufferers [13]. Boardman and colleagues published longitudinal data showing that insomnia preceded and predicted new onset headache and the exacerbation of migraine [14, 15]. Conversely, migraine headaches are also a precursor to insomnia [16].

The main goals of the acute treatment of migraine include rapidly treating the attack with minimal recurrence, reducing the use of additional rescue medications, restoring function, minimizing subsequent resource use, being cost-effective and minimizing the occurrence of adverse events [17, 18]. However, it is often overlooked how important mood disorders and mental health are to the recovery and daily life of migraine sufferers. At present, the pathogenesis of migraine is not yet clear. Scholars consider it to be mostly related to neurovascular factors among others. Drug treatment is commonly used in clinical practice, such as non-steroidal anti-inflammatory drugs (NSAIDs), barbiturates and opioids, but the efficacy is not good and adverse reactions often occur [19], and there are no specific medications to improve mental health throughout the course of illness. Acupuncture treatment for this disease has a long history; it is safe and effective and has been widely recognized in clinical practice [20]. The latest clinical guidelines of Western medicine also recommend acupuncture for the prevention and treatment of migraine [21, 22]. Compared with medication, acupuncture therapy is more cost-effective and has fewer side effects, making it an attractive option for the auxiliary regulation and prevention of various chronic diseases [23]. The goals of acupuncture treatment are usually two-fold: to relieve pain during a migraine attack and to prevent future migraine attacks [24, 25]. In addition, acupuncture can effectively relieve migraine, adjust the mental and psychological state of patients and improve the depression/anxiety disorder of migraine patients [26, 27]. Acupuncture was also supported by relevant studies in improving other concomitant symptoms and quality of life of migraine patients. For example, regulating social functions, role emotions and improving the quality of life all have certain promoting effects [28–31].

Although there is evidence that acupuncture can prevent and relieve migraine and concomitant symptoms such as depression and sleep disturbances, several long-term follow-ups of high-quality RCTs indicate that acupuncture improves the frequency of migraine attacks and the length of the headache, but do not specify whether the long-term follow-up after treatment could improve the mental health of patients [20, 28, 30–32]; therefore, the long-term effectiveness of acupuncture in improving mental health in migraine patients is unclear. In order to better understand the effectiveness of acupuncture for migraine and its impact on patients’ quality of life and related mental health, including pain and mood disorders, we conducted a systematic review and meta-analysis of randomized controlled trials (RCTs) of acupuncture for migraine.

## Method

The study was conducted in accordance with the Preferred Reporting Items for Systematic Reviews and Meta-Analyses (PRISMA) [33] and complied with the PRISMA check list. It was approved by the International Prospective Register of Systematic Reviews (PROSPERO) on February 5, 2022, with the registration number: CRD42022300712.

### Search strategy 

PubMed, Cochrane Library, Embase, Web of Science, Chinese National Knowledge Infrastructure (CNKI) and Wan Fang Data Knowledge Service Platform were visited to identify randomized controlled trials of acupuncture in the treatment of migraine; the supplementary literature was manually searched. Search terms were as follows: “headache”, “migraine”, “cephalgia”, “cephalalgia”, “acupuncture”, “manual acupuncture”, “electro-acupuncture”, “auricular acupuncture”, “migraine and acupuncture”. All search dates were completed by December 31, 2021, we included RCTs published before January 1, 2021.

### Types of study selected 

The studies were RCTs or controlled clinical trials on acupuncture for migraine, with or without blinding or allocation concealment. The following articles were excluded: (1) case reports, reviews and animal studies, (2) repeated experiments, (3) no clear diagnostic criteria for migraine, irregular patient assessment and (4) During the treatment, the experimental group used a treatment other than acupuncture (such as acupuncture combined with traditional Chinese medicine or moxibustion).

### Types of patients included 

Patients were diagnosed as “migraine” under the internationally recognized diagnostic criteria for migraine, regardless of age, sex, course and case origin; no other diseases were included.

### Types of intervention included 

The experimental group was treated with acupuncture, including traditional acupuncture, electro-acupuncture, ear acupuncture, warm acupuncture, etc. while the control group was treated with sham acupuncture or drugs. The details of the acupuncture method are clearly explained, including the selection of needles, acupoints, operations and treatment processes, according to the Intervention Standards for Acupuncture Clinical Experiment Reports [34].

### Types of outcomes 

Since the purpose of this study was to investigate the effects of acupuncture on anxiety, depression and other emotional disorders in migraine patients, the main outcome indicators such as Pain-Related Mood Disorders (Zung Self-Rated Anxiety Scale (SAS), Zung Self-rating Depression Scale (SDS) and Short Form 36 Mental Health (MH) that can better reflect the improvement of mood were selected as the main outcome indicators. Although we focused more on mood disorders associated with migraines, pain and quality of life scores in migraine patients were also of concern, so we chose Visual Analog Scale (VAS) scores for acupuncture migraine and Migraine-Specific Quality of Life Questionnaire (MSQ) emotional Functioning Subscaleas secondary outcomes.

### Data extraction 

Two independent reviewers performed data extraction. Extracted data included study design, diagnostic criteria, migraine duration, sample size, age, gender of participants, intervention, control, duration of treatment, duration of follow-up and adverse events. Incomplete data or inquiries were followed up with the original authors by phone and email. 

### Risk of bias 

We assessed the risk of bias in the included literature using Cochrane RoB2.0 [35]. Each randomized controlled trial was evaluated according to the following six items:(1) the randomization process; (2) Interventions that deviate from expectations; (3) Lack of result data; (4) Measurement results; (5) Select report results; (6) Overall. If the methodology used is appropriate and properly and clearly described, the study is considered low risk; Otherwise, if the method cannot be accurately judged, it is rated as high risk, or there are some problems. Two investigators independently assessed these factors and, if necessary, consulted a third investigator (SY) to resolve differences.

### Statistical analysis

Two researchers performed statistical analysis of the data using Stata 16.0 software. Dichotomous variables are shown as risk ratios (RR) and corresponding 95% CIs. Continuous data were expressed as weighted mean differences (WMD) or standardized mean differences (SMD) and corresponding 95% CI values, with reference to I^2^ and *P* values to determine whether there was heterogeneity in the assessment statistics. If I^2^ > 50% or *P* < 0.01, indicating significant heterogeneity, a random effects model was employed, otherwise, a fixed effects model was used. If there was significant heterogeneity, subgroup analysis or meta-regression was performed to discover sources of heterogeneity. In the statistical description, *P* < 0.05 was considered statistically significant. In addition, a sensitivity analysis was performed to test the soundness of the results by excluding low-quality trials. Funnel and Egger plots were used to detect publication bias and when significant publication bias was found, a trim-fill method was used to test the stability of the results.

## Results 

**Fig. 1:**
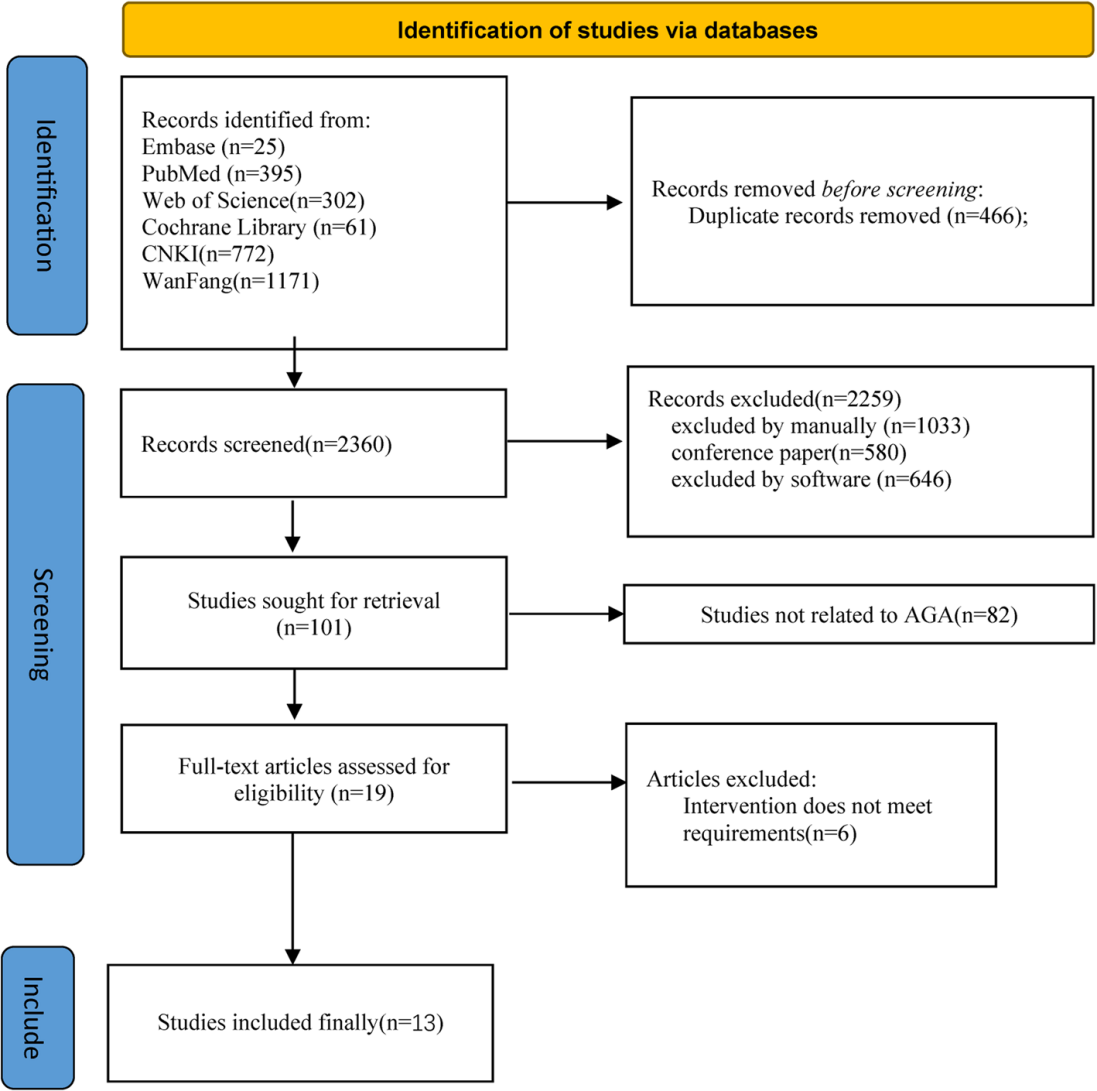
Flow diagram of the included and excluded studies in the systematic review

A total of 2826 reports were retrieved, all published in journals; in total, 466 duplicates were excluded and 580 reports in conference papers were excluded. 1679 articles were excluded, of which 1033 were manually excluded and 646 were excluded by software.82 articles were excluded because the title and abstract indicated that the articles were not related to migraine and 6 articles were excluded after further reading because the efficacy criteria and timing of evaluation of acupuncture treatment did not meet the inclusion requirements. Subsequent analyses included 13 articles (Fig. 1).

### Basic characteristics of eligible studies 

Main characteristics of included studies are shown in Table 1: study place, sample size of treatment and control groups, treatment method selected for treatment group, treatment methods used in control group, time course of efficacy assessment, efficacy assessment indicators, use of acute medication and follow-up period, and adverse events.

**Table 1:** The characteristics of included studies

Study	Country	Outcome measures	Experimental treatment	Control treatment	Sample size (female/male) (E/C)	Age(y) [mean(SD)] (E/C)	Duration(M)	Rescue medication	Adverse events(E/C)
Diener2006 [28]	Germany	③	Verum acupuncture	sham acupuncture	247/43 /257/60	37.1(10.5) /38.3(10.4)	6.5	Beta blockers,fl unarizine,or valproic acid	5/5
Ferro2012 [29]	Brazil	③④	acupuncture	Tanacetum	22/23	38.2(7.4) /37.3(8.6)	2.5	-	-
Guan2018 [36]	China	③④	Acupuncture	Flunarizine hydrochloride	41/24 /47/18	45.9(10.3) /46.4(10.2)	3	-	-
Li 2012 [37]	China	④⑤	acupuncture	sham acupuncture	100/21 /103/15	37.1(11.7) /37.5(12.1)	4	ibuprofen	9(③④⑤) /8(⑨⑩)
Li 2017 [38]	China	①②④	Verum acupuncture	sham acupuncture	9/2 /9/2	21.73(1.98) /21.18(1.12)	1	ibuprofen	-
Wang2011 [30]	China	③④	verum acupuncture	flunarizine	59/11 /60/10	39.2(10.9) /39.2(10.9)	4		5(④⑦⑧) /7(②⑪⑫)
WU2011 [39]	China	③	Acupuncture	Flunarizine hydrochloride	21/9 /19/11	39.6(9.7) /39.1(10.2)	1	ibuprofen	-
Xiao2018 [40]	China	①②④	Electric acupuncture	Diclofenac sodium enteric-coated	19/11 /16/14	30.8(11.2) /35.9(9.6)	0.3	-	-
Xu2020 [32]	China	④⑤	Manual acupuncture	sham acupuncture	47/13 /50/10	36.3(12.0) /36.0(10.9)	5	Diclofenac sodium enteric coated tablets	5/0
Yang2011 [31]	China Taiwan	③	Acupuncture	topiramate	30/3 /29/4	47.6(7.4) /48.1(6.4)	3		2(②⑥⑨) /22(④⑪⑬⑭⑮⑯⑰)
Zhang2021 [41]	China	①②④	Acupuncture	Shame acupuncture	24 /20	33.04(6.43) /35.3(9.43)	3		
Zhao2017 [42]	China	①②④⑤	truth acupuncture	sham acupuncture	65/18 /63/17	36.4(14.2) /39.1(14.6)	6	ibuprofen	5(③⑩)/2(⑨)
Zhuang2017 [43]	China	①②	Manual acupuncture	Carbamazepine tablets	27/18 /26/19	37.5(4.7) /37.8(4.3)	1	-	-

Outcome measures: ①Zung Self-Rated Anxiety Scale (SAS); ②Zung Self-rating Depression Scale (SDS); ③Short Form 36 Mental Health; ④visual Analog Scale scores (VAS); ⑤Migraine-Specific Quality of Life Questionnaire Emotional Functioning Subscale (MSQ)

Adverse events:

Experimental: ①sedation; ②local pain; ③Subcutaneous hemorrhage; ④subcutaneous hematoma; ⑤leg weakness; ⑥subcutaneous ecchymosis; ⑦discomfort; ⑧fatigue; ⑨local paresthesia; ⑩a tingling sensation

Control: ①drowsiness; ②weight gain; ③depression; ④nausea; ⑤constipation; ⑥abdominal pain; ⑦drowsiness; ⑧itching; ⑨subcutaneous hemorrhage; ⑩subcutaneous hematomash; ⑪fatigue; ⑫ faintness; ⑬paresthesia; ⑭difficulty with memory; ⑮dyspepsia; ⑯dizziness; ⑰somnolence

Thirteen randomized controlled trials included 1766 patients: 884 received acupuncture, 296 served with a drug control, and 606 were treated by sham acupuncture. The treatment groups in the 13 included studies were all acupuncture alone. Regarding the types of control groups included in the analysis, 6 used sham acupuncture [20, 28, 32, 37, 38, 41], 7used drug [29–31, 36, 39, 40, 43], Six [20, 28, 30, 32, 36, 37] of the 13 studies were multicenter controlled trials, and the rest were single-center controlled trials (Fig. 1).

For the diagnosis of migraine, 13 studies used internationally recognized diagnostic criteria, and for migraine efficacy criteria. 5 studies used SAS and SDS scales [20, 38, 40, 41, 43], 6 studies [28–31, 36, 39]used the short form(SF)mental health.9 studies used VAS to measure the intensity of visual pain presented by patients [20, 29, 30, 32, 37–41].and3 studies used the MSQ to measure patients' emotional function [20, 32, 37]. 6 reported adverse events [20, 28, 30–32, 37].

### Risk of bias

**Fig. 2:**
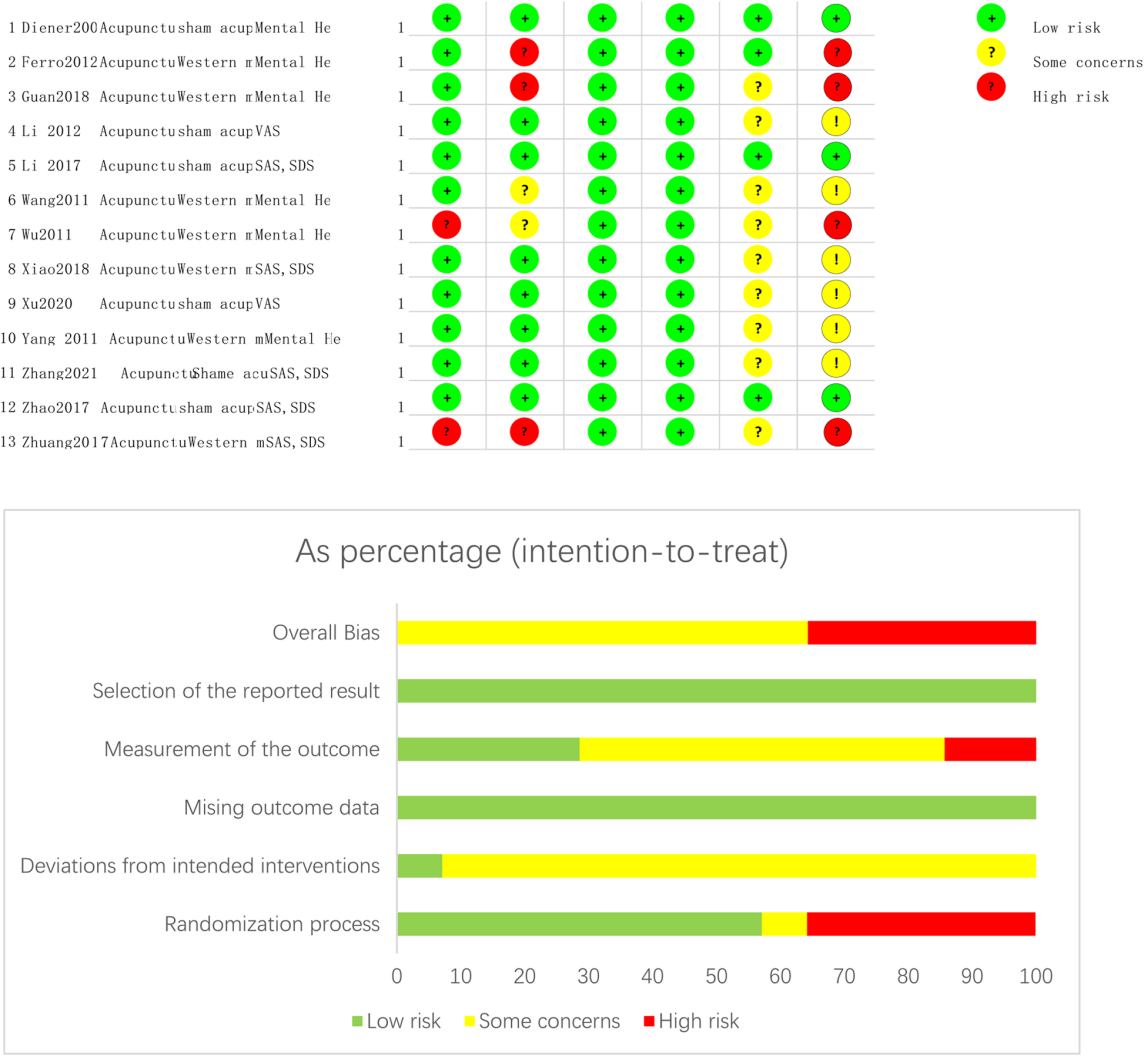
Risk of bias graph

Nine RCTS had a moderate or low risk of bias, and two [39, 43] RCTS had a high risk of bias due to randomisation. Six cohort studies had a moderate risk of bias and four had a severe risk of bias (Fig. 2).

### Primary outcomes

#### SAS 

**Fig. 3:**
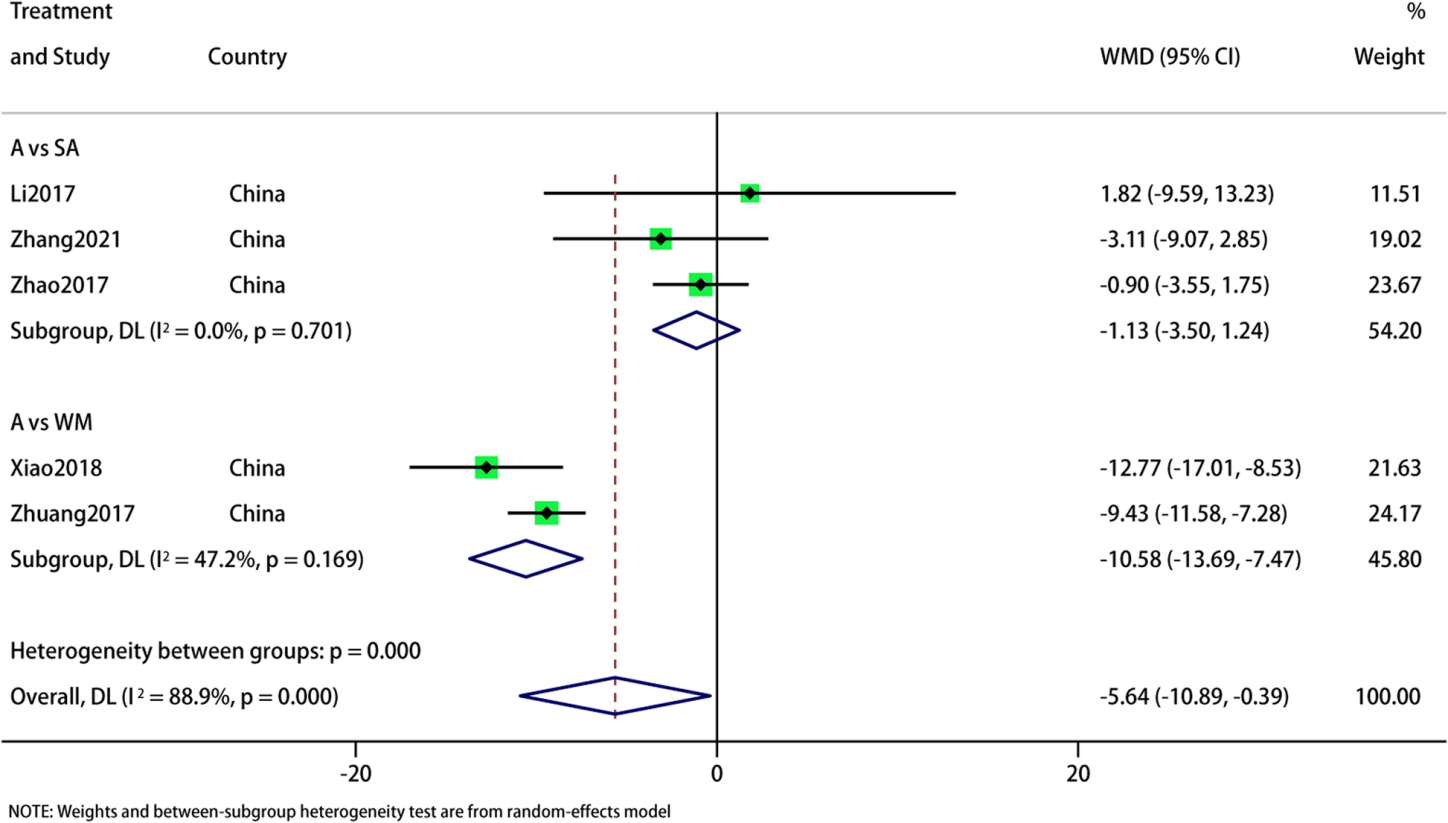
Meta-analysis of SAS score with migraine

**Fig. 4:**
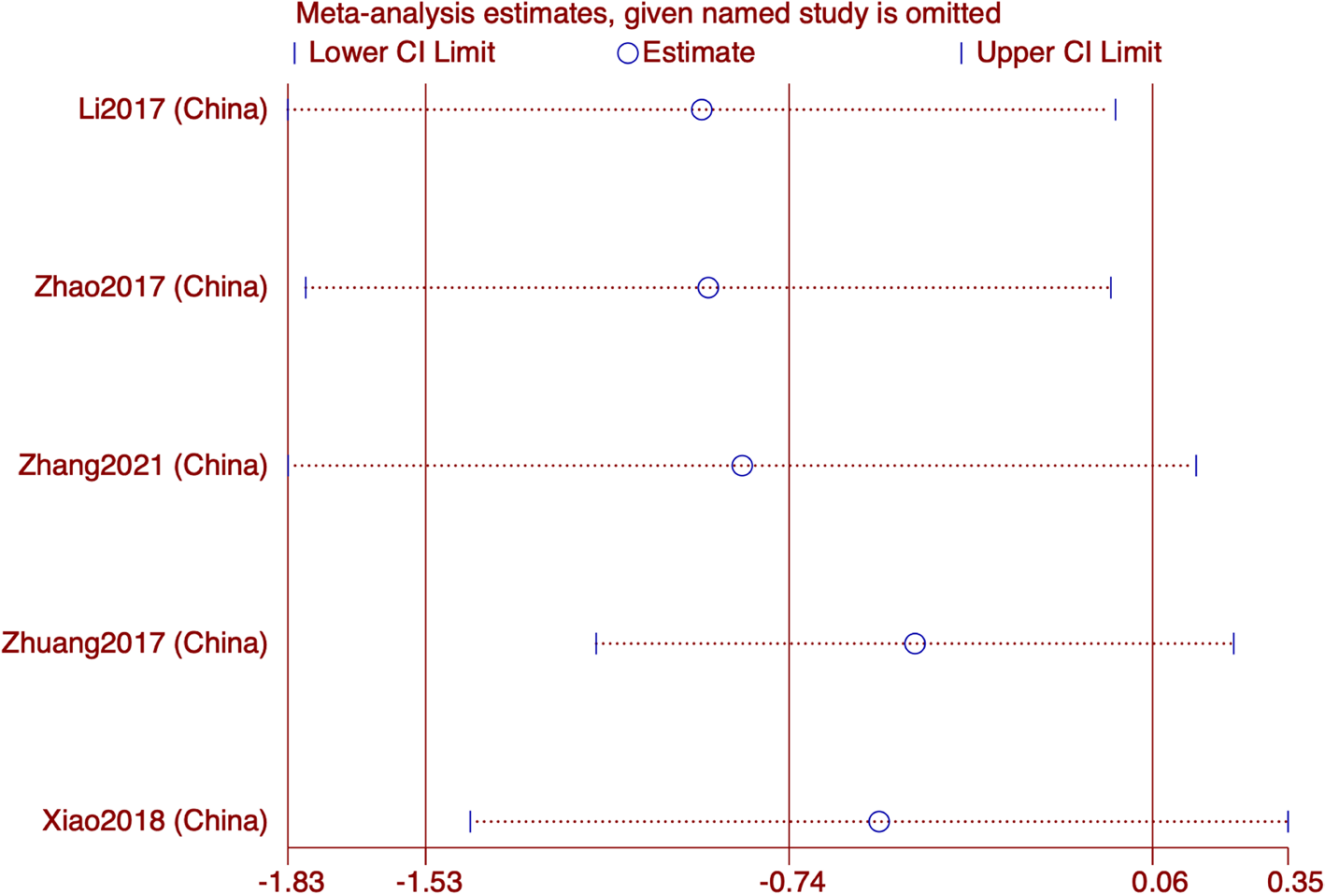
SAS analysis of sensitivity

Five RCTs reported the SAS. Subgroup analysis showed no difference between acupuncture and sham acupuncture (WMD: -1.13;95% CI: -3.5, 1.24; *p* = 0.349) (I^2^ = 0%, *p* = 0.701) in improving SAS, but superior to Western medicine (WMD: -10.58;95% CI: -13.69, -7.47; *p* < 0.0001) (I^2^ = 47.2%, *p* = 0.169). Overall, the difference was statistically significant, (WMD: -5.64;95% CI: -10.89, -0.39;*p* = 0.035).and heterogeneity was significant (I^*2*^ = 88.9%, *p* = 0), and hence a fixed-effects model was used to merge the data (Fig. 3). The results of sensitivity analysis were stable (Fig. 4). The GRADE quality of this evidence is very low (Table 2). As the number of RCTs on outcome indicators is less than 10, it is not enough to publication bias analysis.

**Table 2:** The grade evidence quality of acupuncture or migraine

Quality assessment							No of patients		Effect		Quality	Importance
**No of studies**	**Design**	**Risk of bias**	**Inconsistency**	**Indirectness**	**Imprecision**	**Other considerations**	**Acupuncture**	**Control**	**Relative (95% CI)**	**Absolute**		
**SAS (Better indicated by lower values)**												
5	randomised trials	no serious risk of bias	Serious^a^	Serious^b^	very serious^c^	none	193	186	-	MD 5.64 lower (10.89 to 0.39 lower)	ÅOOO VERY LOW	IMPORTANT
**SAS—A vs SA (Better indicated by lower values)**												
3	no methodology chosen					none	118	111	-	MD 1.13 lower (3.5 lower to 1.24 higher)		
**SAS—A vs WM (Better indicated by lower values)**												
2	no methodology chosen					none	75	75	-	MD 10.58 lower (13.69 to 7.47 lower)		
**VAS (Better indicated by lower values)**												
9	randomised trials	no serious risk of bias	Serious^a^	Serious^b^	very serious^c^	none	479	471	-	MD 1.06 lower (1.72 to 0.39 lower)	ÅOOO VERY LOW	
**VAS—A vs SA (Better indicated by lower values)**												
6	no methodology chosen					none	367	359	-	MD 0.89 lower (1.76 to 0.02 lower)		
**VAS—A vs WM (Better indicated by lower values)**												
3	no methodology chosen					none	112	112	-	MD 1.39 lower (2.29 to 0.49 lower)		
**SDS (Better indicated by lower values)**												
5	randomised trials	no serious risk of bias	Serious^a^	Serious^b^	very serious^c^	none	99	95	-	MD 4.65 lower (9.25 to 0.045 lower)	ÅOOO VERY LOW	
**SDS—A vs SA (Better indicated by lower values)**												
3	no methodology chosen					none	24	20	-	MD 0.72 lower (3.76 lower to2.23 higher)		
**SDS—A vs WM (Better indicated by lower values)**												
2	no methodology chosen					none	75	75	-	MD 9.5 lower (15.6 to3.4 lower)		
**SF-MH (Better indicated by lower values)**												
6	randomised trials	no serious risk of bias	Serious^a^	Serious^b^	very serious^c^	none	505	532	-	MD 3.87 higher (1.49 to 6.25 higher)	ÅOOO VERY LOW	
**SF-MH—A vs SA (Better indicated by lower values)**												
2	no methodology chosen					none	360	387	-	MD 1.42higher (1.69 lower to 4.54 higher)		
**SF-MH—A vs WM (Better indicated by lower values)**												
4	no methodology chosen					none	145	145	-	MD5.3 higher (1.82 to 8.78 higher)		
**MSQ (Better indicated by lower values)**												
3	randomised trials	no serious risk of bias	Serious^a^	Serious^b^	serious	none	262	258	-	MD 0.57 higher (0.21 to 0.93 higher)	ÅOOO VERY LOW	IMPORTANT

^a^The 95% confidence interval crosses the equivalence line

^b^Two-thirds come from medium bias

^c^I^2^ > 75%

#### SDS 

**Fig. 5:**
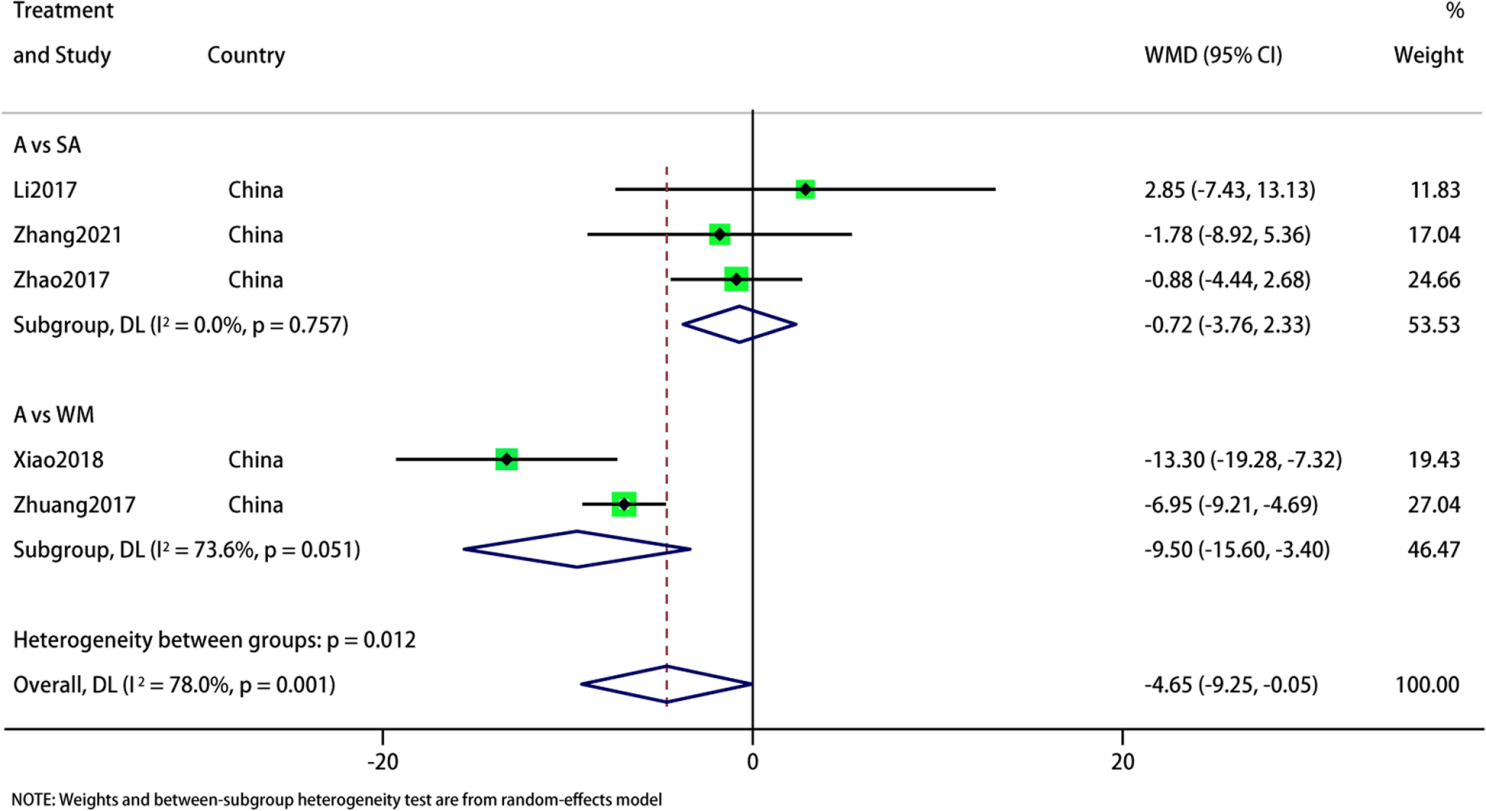
Meta-analysis of SDS score with migraine

**Fig. 6:**
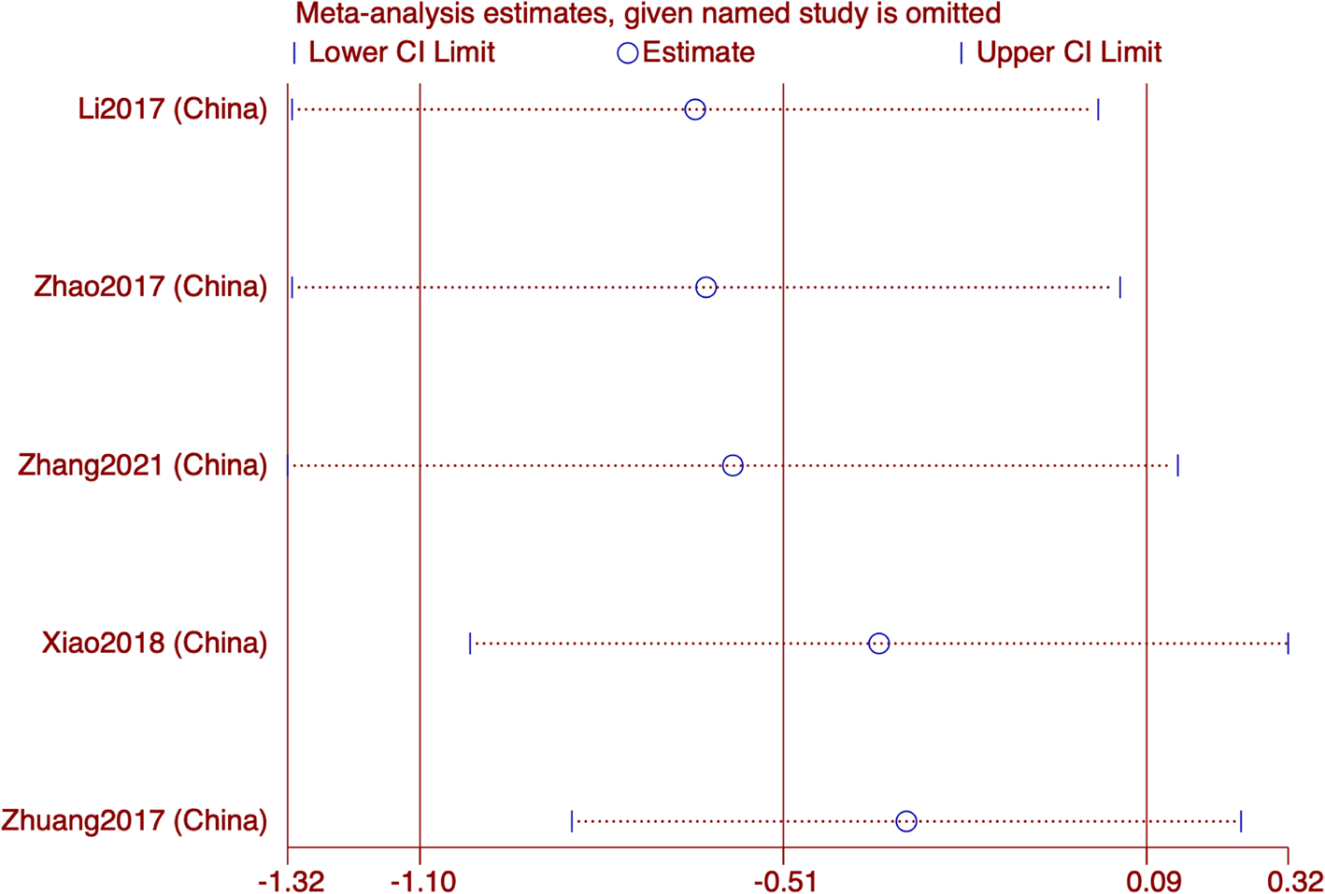
SDS analysis of sensitivity

In the five studies that reported SDS, we did a subgroup analysis based on the intervention of the control group. The pooled results showed that the current results show that acupuncture is superior to sham acupuncture and Western medicine in improving SDS in migraine patients (WMD: -4.65; 95% CI: -9.25, -0.05; *p* = 0.048), and the heterogeneity is significant (I^*2*^ = 78%, *p* = 0.001). The subgroup showed high heterogeneity and no source of heterogeneity was found (Fig. 5). The results of sensitivity analysis were stable (Fig. 6). The GRADE quality of this evidence is very low.

#### MH

**Fig. 7:**
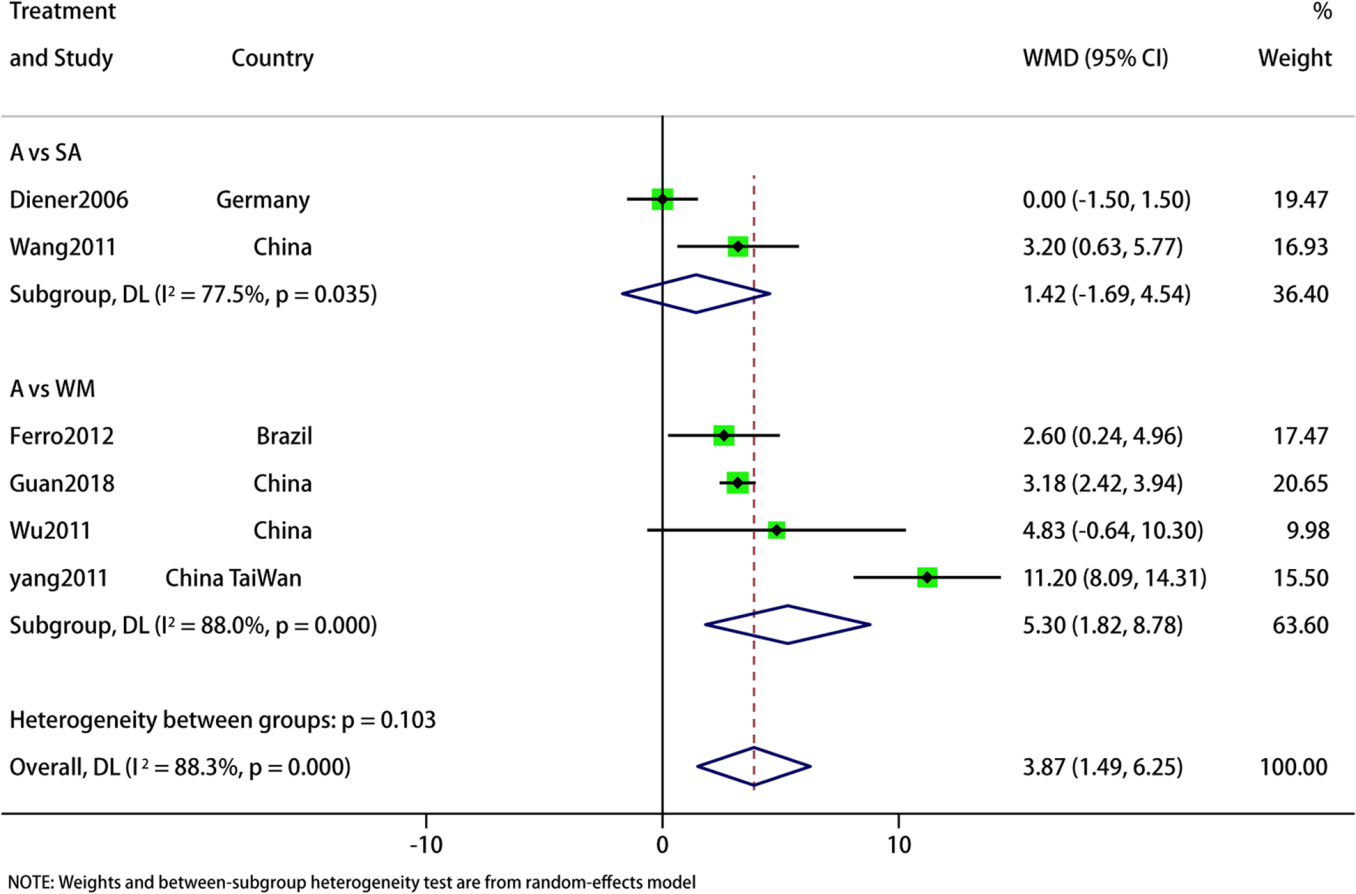
Meta-analysis of mental health (MH) score with migraine

**Fig. 8:**
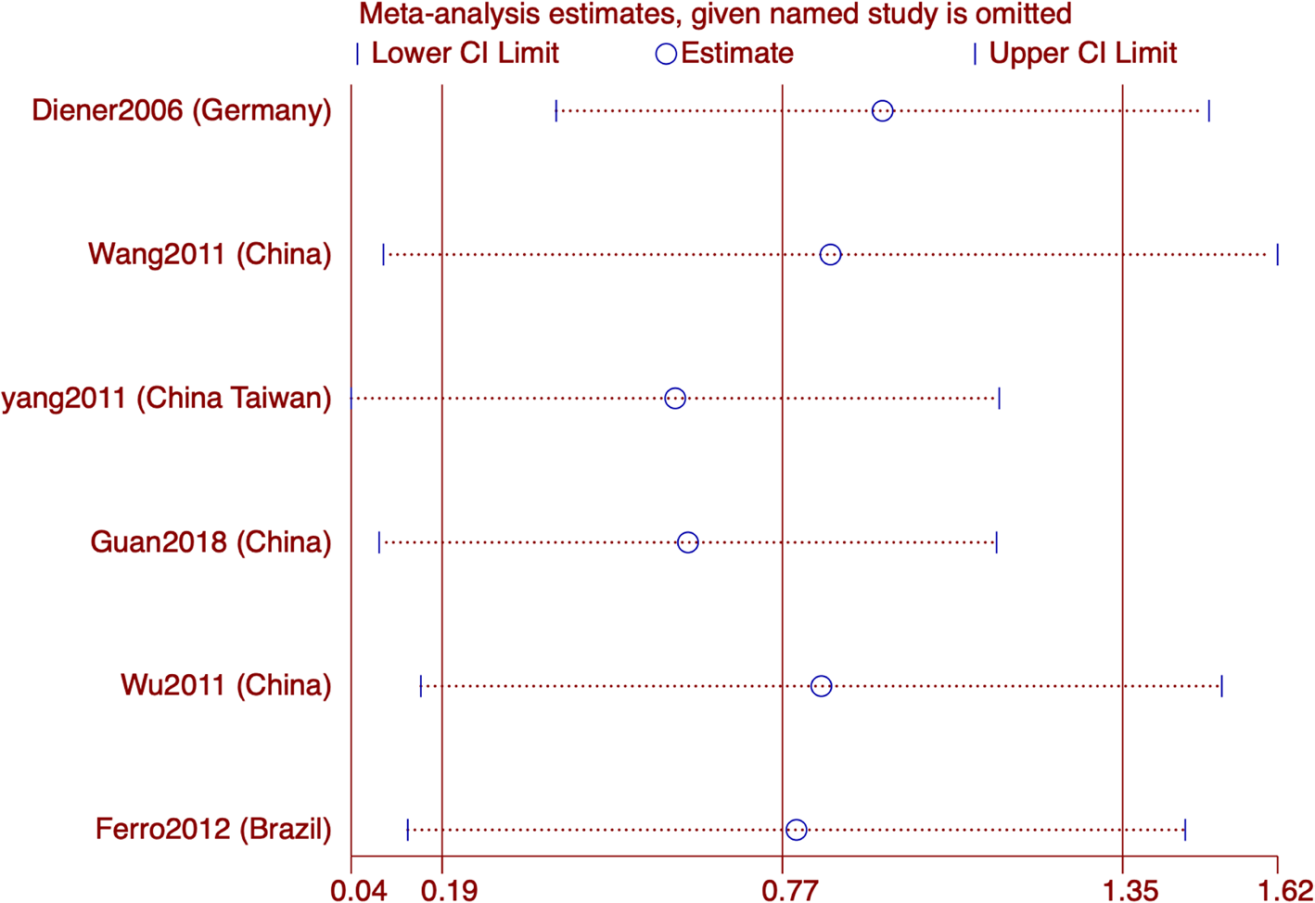
MH analysis of sensitivity

Six articles reported MH, and the aggregated results of subgroup analyses showed that acupuncture improved MH in migraine patients better than the control group (WMD: 3.87; 95% CI: 1.49, 6.25; *p* = 0.001). But with high heterogeneity (I^2^ = 88.3%, *p* = 0). The subgroup showed high heterogeneity and no source of heterogeneity was found. The GRADE quality of this evidence was very low. The results of sensitivity analysis is stable (Figs. 7 and 8).

### Secondary outcomes

#### VAS

**Fig. 9:**
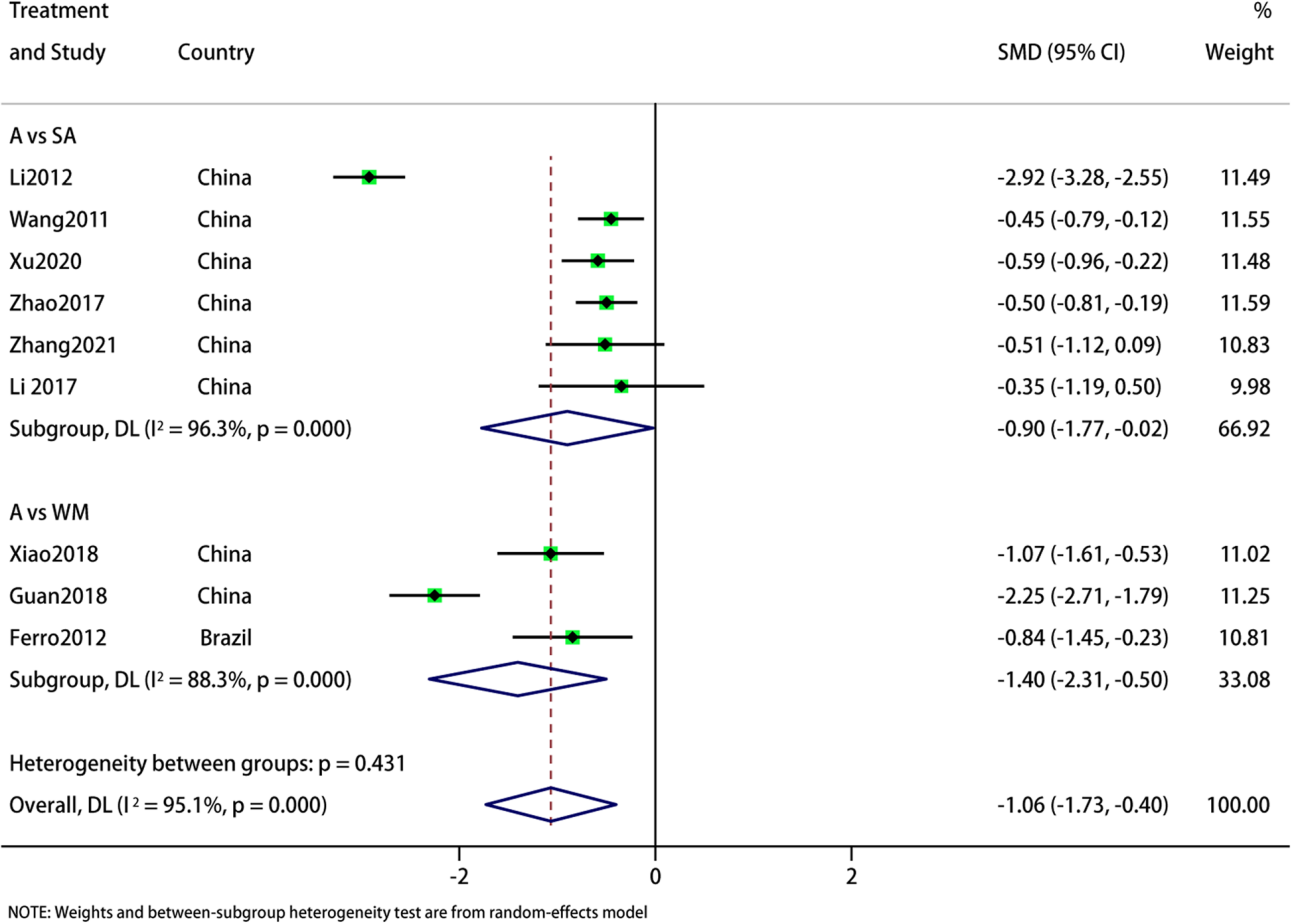
Meta-analysis of VAS score for migraine

**Fig. 10:**
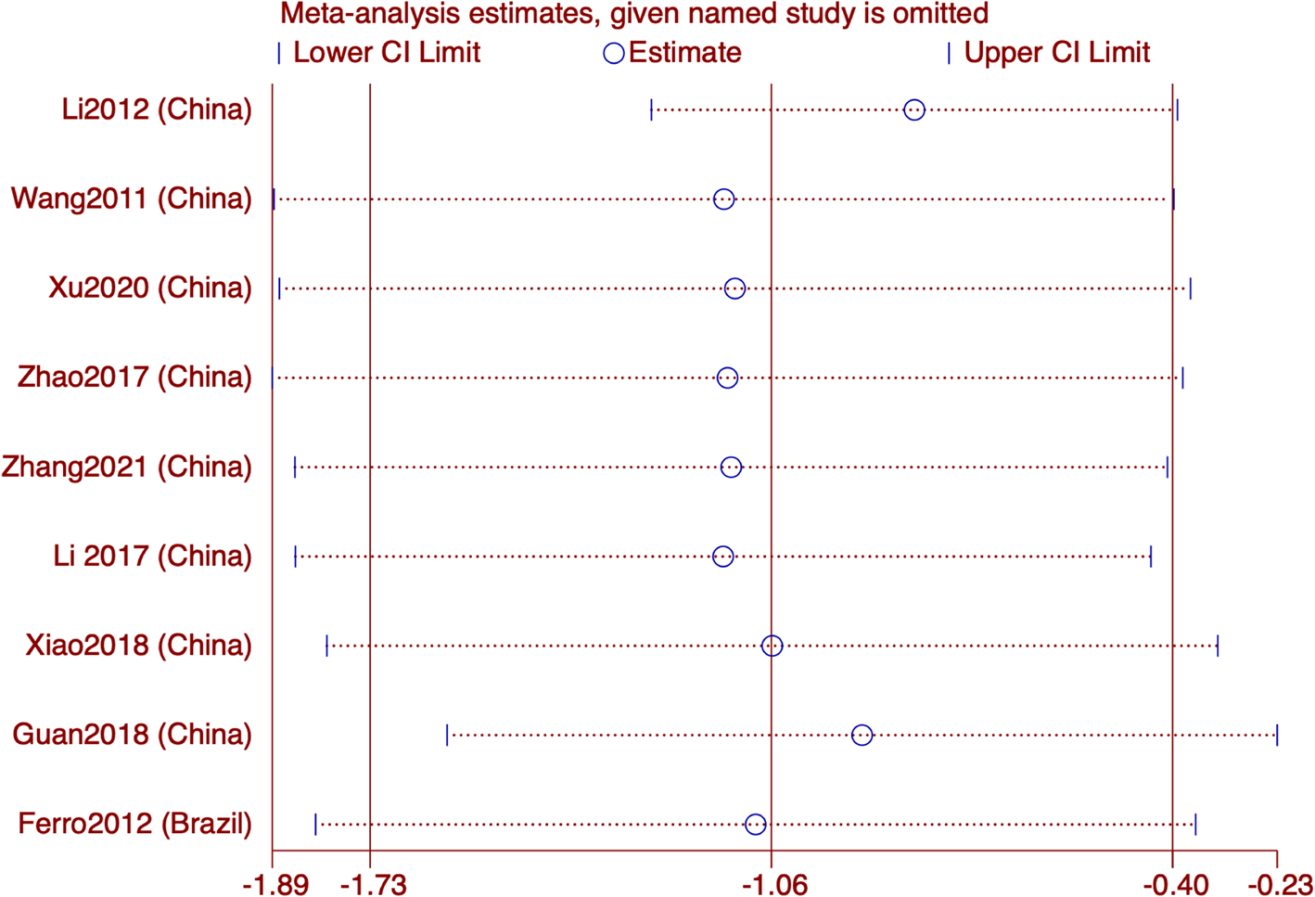
VAS analysis of sensitivity

In 9 RCTS reporting VAS, subgroup analysis showed that acupuncture was superior to sham acupuncture (SMD: -0.9; 95% CI: -1.77, -0.02; *p* = 0.002) and Western medicine (SMD: -1.4; 95% CI: -2.31, -0.5; *p* = 0.002;) in improving VAS. The summary results are (SMD: -1.06; 95% CI: -1.73, -0.4; *p* = 0.002;) Sensitivity analysis showed that the results were stable. However, forest map results showed significant heterogeneity in both subgroups and total results (I^2^ = 95.1%, *p* = 0.000), and the source of heterogeneity was uncertain. The GRADE quality of this evidence is very low (Figs. 9 and 10).

#### MSQ

**Fig. 11:**
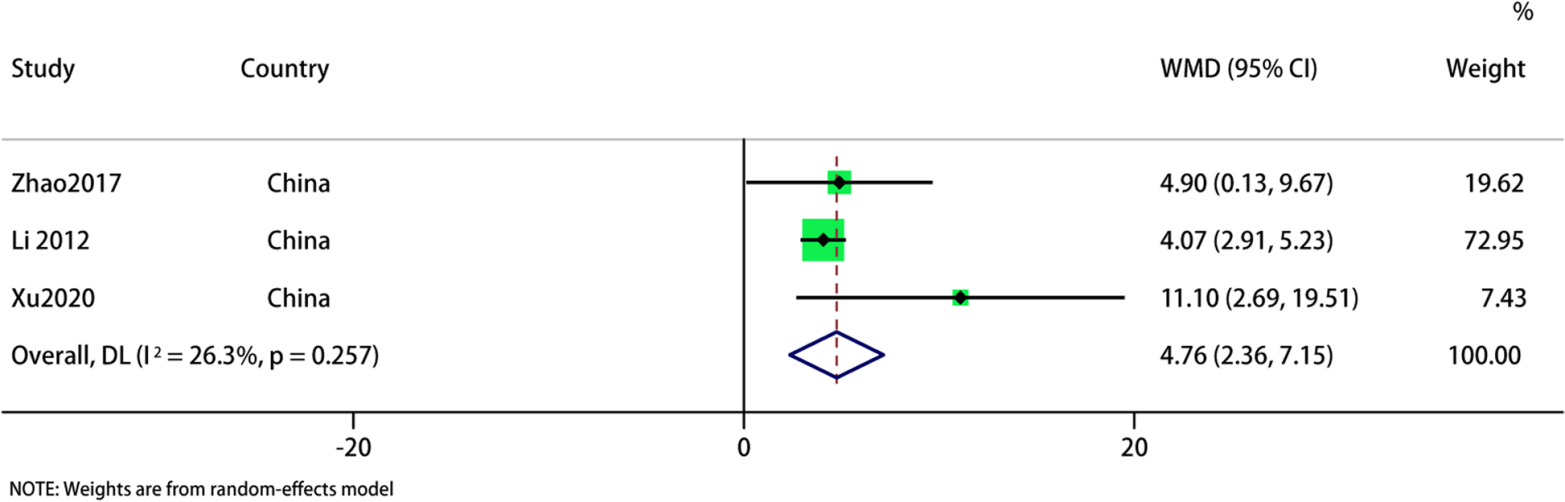
Meta-analysis of MSQ score for migraine

**Fig. 12:**
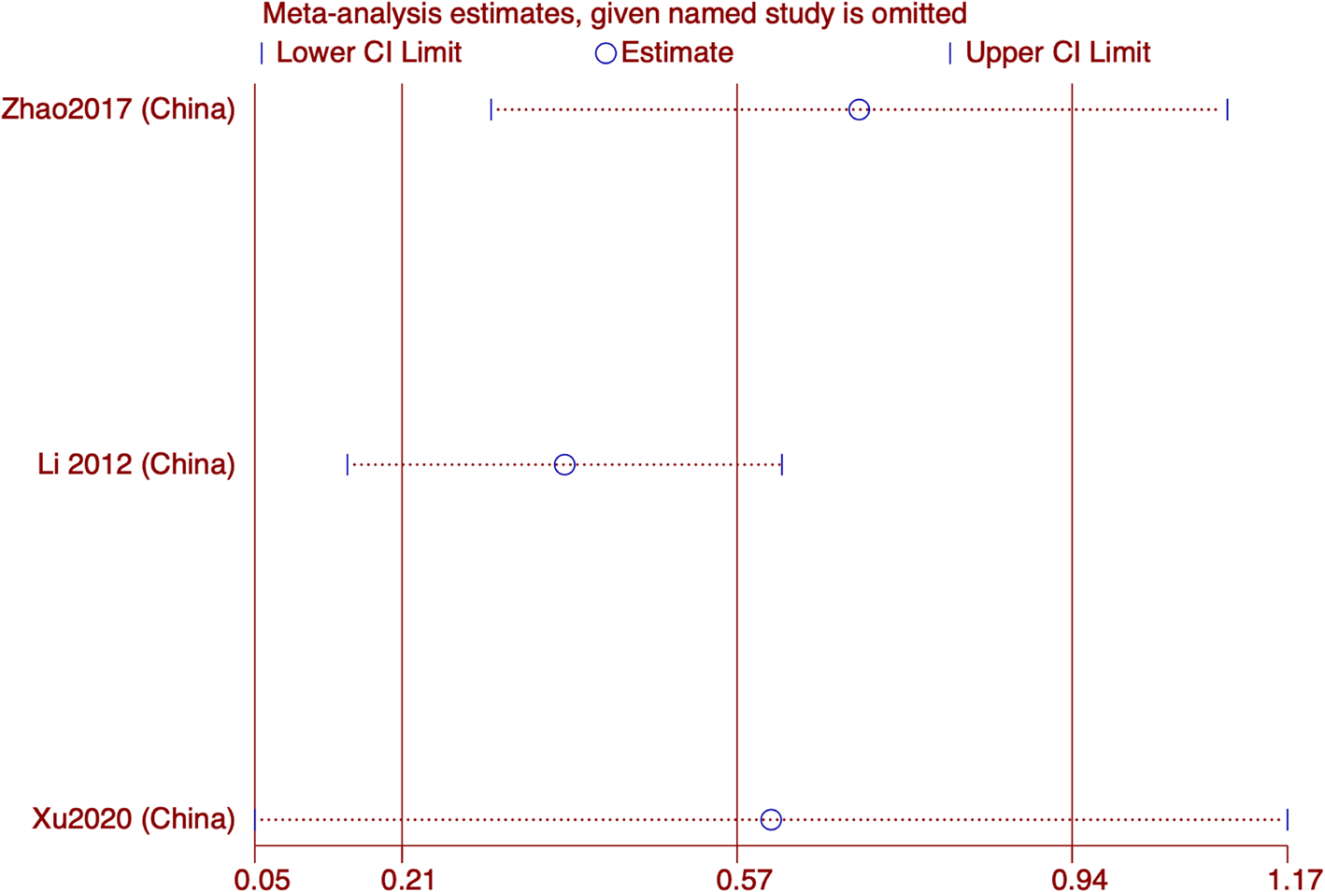
MSQ analysis of sensitivity

Only 3 studies reported MSQ results, and the forest map showed that acupuncture was superior to sham acupuncture in improving MSQ in migraine patients (WMD: 4.76; 95% CI: 2.36, 7.15; *p* < 0.001). The heterogeneity is not significant (I^2^ = 26.3%; *p* = 0.257). Sensitivity analysis showed that the results is stable (Figs. 11 and 12).

## Discussion 

This study aimed to evaluate the efficacy of acupuncture in improving the mental health of migraine patients. We found that the experimental group and the control group had statistical significance in improving SAS, SDS MH, VAS and MSQ in migraine patients.

The pathogenesis of migraine is not fully understood and its pathogenesis involves complex pathophysiological changes [44]. Migraine attacks can be accompanied by a variety of neurological, gastrointestinal and autonomic changes [45] and should not be considered a vascular headache alone [46]. Some scholars have proposed that migraine is triggered by activation of the trigeminal sensory pathway [47]. The basis of the characteristic symptoms of migraine may be related to auditory, visual and olfactory cortical areas receiving trigeminal sensory input, while somatosensory, insular, retrosplenic and parietal relevant cortical areas are closely related to sensory discrimination, emotional and cognitive evaluation of trigeminal nociceptive input [48]. Interictal calcitonin gene-related peptide (CGRP) and vasoactive intestinal peptide (VIP) levels in patients with chronic migraine were higher than those in patients with episodic migraine [49], suggesting that the interictal activities of the trigeminal nerve and cranial autonomic nervous system changed in patients with chronic migraine.

Chronic migraine is not life-threatening, but can seriously affect the patient’s quality of life. Studies have shown that migraine patients are more likely to suffer from anxiety and depression than healthy people [50] and the risk of disability is greatly increased. Current first-line drugs for migraine include divalproex, topiramate, metoprolol, propranolol and timolol [51]. However, in the prophylactic treatment of migraine and its associated symptoms, the administration of a single drug to treat both conditions may not be effective and optimal [52], so a combination of drugs is often required and taken over a long period of time. As a supplementary therapy for migraine, acupuncture is well tolerated and has few adverse reactions, so it is widely used in clinical practice [53]. Numerous studies have also confirmed that acupuncture is an effective method for the treatment of migraine [54].

Regarding the mechanism of acupuncture in the treatment of migraine, some scholars have conducted animal experiments and studies have shown that electroacupuncture significantly improves mechanical and thermal hyperalgesia, reduces c-Fos levels in trigeminal ganglia and decreased levels of vasoactive neurotransmitters in plasma and dura mater [55]. Electroacupuncture can improve migraine and related cutaneous hypersensitivity reactions by modulating the ascending pathway of the trigeminal vasculature, partly by inhibiting the expression of CGRP in the trigeminal ganglion [42] and by increasing the receptors in the periorbital region of the trigeminal nerve. Other studies have shown that electroacupuncture inhibits hyperalgesia induced by dural electrical stimulation (DES) by reducing inflammatory factors. The inhibition of dural mast cells, macrophages and serum inflammatory factors may be one of the mechanisms of acupuncture in the treatment of migraine [56]. There are also clinical studies showing that acupuncture can significantly reduce matrix metalloproteinase-2 (MMP-2) in migraine patients [57]. Therefore, it is speculated that the mechanism of acupuncture to relieve pain may be related to the reduction of MMP-2. In addition, acupuncture can relieve headache by modulating the neural activity of related brain regions by regulating the frontal, parietal and limbic regions of the brain for specific therapeutic effects and non-specific analgesics [58].

The interventions tested in the RCTs in 13 studies were all acupuncture and we did not differentiate between acupoint selection and manipulation, which may have contributed to heterogeneity. In a broad sense, electro-acupuncture, warm acupuncture, ear acupuncture and body acupuncture belong to acupuncture and moxibustion, but the related literature on ear acupuncture, electro-acupuncture and other treatments were excluded because they were inconsistent with the purpose of this study. Studies on acupoint injection and acupoint pressing were excluded because acupoint pressing belongs to massage, while acupoint injection is often conducted with drugs and does not belong to the scope of acupuncture and moxibustion. The control group received sham acupuncture or drugs. The drug group used different drugs, including flunarizine, valproic acid, tanasedone and topiramate. However, because the sample size of the included literature is small and the doses of different drugs are inconsistent, the main observation indicators in this paper focus is on the psycho-emotional effects of acupuncture in patients with migraine. It is well known that sham acupuncture and drugs are treated in significantly different ways, so we conducted a subgroup analysis of different interventions in the control group. This may be the main reason for the significant heterogeneity of results.

There are some limitations to our study: first, there was large heterogeneity between analyses. This may be related to the inconsistency of acupoint selection, treatment time and follow-up time; the difference in sample size may also be one of the important factors affecting the results. There are studies showing that the differences in the effects of various acupuncture experiments are mainly driven by the differences in the treatments received by the control group, rather than differences in the characteristics of acupuncture treatments [59]. Secondly, it is extremely difficult to reach a complete double-blind experiment. The blindness caused by fake acupuncture and drugs will be suspected by patients, because of the different intervention measures, whether compared with drugs or sham acupuncture, patients easily sense the differences in acupuncture experiments. We believe that the main reason for this is related to patient self-assessment. As the measurement indicators and scores in the results are subjective feelings of the patients, the expected treatment effect and results are different from the experimental results, which may lead to different scores. In addition, patients’ trust in acupuncturists and different techniques of acupuncturists also contribute to the differences in results. After all, a comfortable acupuncture experience is more acceptable to each patient. Moreover, the acupuncture treatment time, ranging from 1 to 12 weeks, and the follow-up time, ranging from 8 to 24 weeks, in the literature are relatively large. Which is more likely to be accepted by doctors or patients is rarely mentioned. Finally, there is no clear generation of random sequences or allocation concealment, resulting in high publication bias.

Therefore, to address potential limitations, we believe that a large-sample, multicenter clinical randomized controlled trial is important and a more rigorous RCT is necessary. Some suggestions include: (1) the definition of long-term and short-term effects should be clarified in acupuncture experiments; (2) the selection of acupoints could be more standardized; (3) appropriate addition and subtraction based on the dialectics of traditional Chinese medicine can more effectively avoid heterogeneity, to improve the comparability of acupuncture and moxibustion with drugs or other treatments and the feasibility of research.

The results of this study show that acupuncture has advantages in treating migraine and improving our quality of life and mental health, so we believe that acupuncture can be a recommended treatment modality. Moreover, the frequency of adverse events of acupuncture is lower and the most common ones are local pain and blood stasis [60], which can disappear naturally after acupuncture stops. Therefore, we recommend acupuncture for migraine.

## Conclusion 

The present results suggest that, compared with Western medicine and sham acupuncture, acupuncture seems to be able to effectively improve anxiety and depression in migraine patients. And it may be more effective in improving SF36-mental health, VAS and MSQ than shame acupuncture and Western medicine. The results of this study need to be verified by higher quality RCTs.

### Supplementary Information

**Additional file 1.**

## Data Availability

All data generated or analysied during this study are included in this published article and in supplementary information files.

## References

[1] GBD 2017 Disease and Injury Incidence and Prevalence Collaborators. Global, regional, and national incidence, prevalence, and years lived with disability for 354 diseases and injuries for 195 countries and territories, 1990-2017: a systematic analysis for the Global Burden of Disease Study 2017. Lancet. 2018;392(10159):1789–858. 10.1016/S0140-6736(18)32279-7. Epub 2018 Nov 8. Erratum in: Lancet. 2019;393(10190):e44. 10.1016/S0140-6736(18)32279-7 PMC6227754 30496104

[2] GBD 2017 Disease and Injury Incidence and Prevalence Collaborators. Global, regional, and national incidence, prevalence, and years lived with disability for 328 diseases and injuries for 195 countries, 1990–2016: a systematic analysis for the Global Burden of Disease Study 2016. Lancet. 2017;390(10100):1211–59. 10.1016/S0140-6736(17)32154-2 PMC5605509 28919117

[3] Buse DC, Manack A, Serrano D, Turkel C, Lipton RB (2010). Sociodemographic and comorbidity profiles of chronic migraine and episodic migraine sufferers. J Neurol Neurosurg Psychiatry.

[4] Ramage-Morin PL, Gilmour H (2014). Prevalence of migraine in the Canadian household population. Health Rep.

[5] Iliopoulos P, Damigos D, Kerezoudi E, Limpitaki G, Xifaras M, Skiada D, Tsagkovits A, Skapinakis P (2015). Trigger factors in primary headaches subtypes: a cross-sectional study from a tertiary centre in Greece. BMC Res Notes.

[6] Kelman L, Rains JC (2005). Headache and sleep: examination of sleep patterns and complaints in a large clinical sample of migraineurs. Headache.

[7] Park JW, Chu MK, Kim JM, Park SG, Cho SJ (2016). Analysis of Trigger Factors in Episodic Migraineurs Using a Smartphone Headache Diary Applications. PLoS One.

[8] Schramm SH, Moebus S, Lehmann N, Galli U, Obermann M, Bock E, Yoon MS, Diener HC, Katsarava Z (2015). The association between stress and headache: A longitudinal population-based study. Cephalalgia.

[9] Swanson SA, Zeng Y, Weeks M, Colman I (2013). The contribution of stress to the comorbidity of migraine and major depression: results from a prospective cohort study. BMJ Open.

[10] Ashina S, Serrano D, Lipton RB, Maizels M, Manack AN, Turkel CC, Reed ML, Buse DC (2012). Depression and risk of transformation of episodic to chronic migraine. J Headache Pain.

[11] Dilsaver SC, Benazzi F, Oedegaard KJ, Fasmer OB, Akiskal HS (2009). Is a family history of bipolar disorder a risk factor for migraine among affectively ill patients?. Psychopathology.

[12] Rains JC (2018). Sleep and Migraine: Assessment and Treatment of Comorbid Sleep Disorders. Headache.

[13] Lee SH, Kang Y, Cho SJ (2017). Subjective cognitive decline in patients with migraine and its relationship with depression, anxiety, and sleep quality. J Headache Pain.

[14] Boardman HF, Thomas E, Millson DS, Croft PR (2005). One-year follow-up of headache in an adult general population. Headache.

[15] Boardman HF, Thomas E, Millson DS, Croft PR (2006). The natural history of headache: predictors of onset and recovery. Cephalalgia.

[16] Ødegård SS, Sand T, Engstrøm M, Zwart JA, Hagen K (2013). The impact of headache and chronic musculoskeletal complaints on the risk of insomnia: longitudinal data from the Nord-Trøndelag health study. J Headache Pain.

[17] Buse DC, Rupnow MF, Lipton RB (2009). Assessing and managing all aspects of migraine: migraine attacks, migraine-related functional impairment, common comorbidities, and quality of life. Mayo Clin Proc.

[18] Silberstein SD (2000). Practice parameter: evidence-based guidelines for migraine headache (an evidence-based review): report of the Quality Standards Subcommittee of the American Academy of Neurology. Neurology.

[19] Moskowitz MA (2007). Pathophysiology of headache–past and present. Headache.

[20] Zhao L, Chen J, Li Y, Sun X, Chang X, Zheng H, Gong B, Huang Y, Yang M, Wu X, Li X, Liang F (2017). The Long-term Effect of Acupuncture for Migraine Prophylaxis: A Randomized Clinical Trial. JAMA Intern Med.

[21] Carville S, Padhi S, Reason T, Underwood M (2012). Diagnosis and management of headaches in young people and adults: summary of NICE guidance. BMJ.

[22] Kennis K, Kernick D, O’flynn N (2013). Diagnosis and management of headaches in young people and adults: NICE guideline. Br J Gen Pract.

[23] Shin S, Yang SP, Yu A, Yoo J, Lim SM, Lee E (2019). Effectiveness and safety of electroacupuncture for poststroke patients with shoulder pain: study protocol for a double-center, randomized, patient- and assessor-blinded, sham-controlled, parallel, clinical trial. BMC Complement Altern Med.

[24] Li Y, Liang F, Yang X, Tian X, Yan J, Sun G, Chang X, Tang Y, Ma T, Zhou L (2009). Acupuncture for treating acute attacks of migraine: a randomized controlled trial. Headache.

[25] Wang L-P, Zhang X-Z, Guo J, Liu H-L, Zhang Y, Liu C-Z, Yi J-H, Wang L-P, Zhao J-P, Li S-S (2012). Efficacy of Acupuncture for Acute Migraine Attack: A Multicenter Single Blinded Randomized Controlled Trial. Pain Medicine.

[26] Liao CC, Liao KR, Lin CL, Li JM (2020). Long-Term Effect of Acupuncture on the Medical Expenditure and Risk of Depression and Anxiety in Migraine Patients: A Retrospective Cohort Study. Front Neurol.

[27] Vijayalakshmi I, Shankar N, Saxena A, Bhatia MS (2014). Comparison of effectiveness of acupuncture therapy and conventional drug therapy on psychological profile of migraine patients. Indian J Physiol Pharmacol.

[28] Diener HC, Kronfeld K, Boewing G, Lungenhausen M, Maier C, Molsberger A, Tegenthoff M, Trampisch HJ, Zenz M, Meinert R (2006). Efficacy of acupuncture for the prophylaxis of migraine: a multicentre randomised controlled clinical trial. Lancet Neurol.

[29] Ferro EC, Biagini AP, Da Silva ÍE, Silva ML, Silva JR (2012). The combined effect of acupuncture and Tanacetum parthenium on quality of life in women with headache: randomised study. Acupunct Med.

[30] Wang LP, Zhang XZ, Guo J, Liu HL, Zhang Y, Liu CZ, Yi JH, Wang LP, Zhao JP, Li SS (2011). Efficacy of acupuncture for migraine prophylaxis: a single-blinded, double-dummy, randomized controlled trial. Pain.

[31] Yang CP, Chang MH, Liu PE, Li TC, Hsieh CL, Hwang KL, Chang HH (2011). Acupuncture versus topiramate in chronic migraine prophylaxis: a randomized clinical trial. Cephalalgia.

[32] Xu S, Yu L, Luo X, Wang M, Chen G, Zhang Q, Liu W, Zhou Z, Song J, Jing H, Huang G, Liang F, Wang H, Wang W (2020). Manual acupuncture versus sham acupuncture and usual care for prophylaxis of episodic migraine without aura: multicentre, randomised clinical trial. Bmj.

[33] Page MJ, Mckenzie JE, Bossuyt PM, Boutron I, Hoffmann TC, Mulrow CD, Shamseer L, Tetzlaff JM, Akl EA, Brennan SE, Chou R, Glanville J, Grimshaw JM, Hróbjartsson A, Lalu MM, Li T, Loder EW, Mayo-Wilson E, Mcdonald S, Mcguinness LA, Stewart LA, Thomas J, Tricco AC, Welch VA, Whiting P, Moher D (2021). The PRISMA 2020 statement: an updated guideline for reporting systematic reviews. Syst Rev.

[34] Macpherson H, Altman DG, Hammerschlag R, Youping L, Taixiang W, White A, Moher D (2010). Revised STandards for Reporting Interventions in Clinical Trials of Acupuncture (STRICTA): Extending the CONSORT statement. J Evid Based Med.

[35] Higgins JPT, Sterne JAC, Savovic J, Page MJ, Hrobjartsson A, Boutron I, Reeves B, Eldridge S (2016). A revised tool for assessing risk of bias in randomized trials. Cochrane Database Syst Rev.

[36] Guan S. Acupuncture "for god six point" the clinical curative effect of treating migraine evaluation studies. Ningxia Medical University. 2018. MA thesis. https://kns.cnki.net/KCMS/detail/detail.aspx?dbname=CMFD201802&filename=1018978153.nh.

[37] Li Y, Zheng H, Witt CM, Roll S, Yu SG, Yan J, Sun GJ, Zhao L, Huang WJ, Chang XR, Zhang HX, Wang DJ, Lan L, Zou R, Liang FR (2012). Acupuncture for migraine prophylaxis: a randomized controlled trial. Cmaj.

[38] Li Z, Zeng F, Yin T, Lan L, Makris N, Jorgenson K, Guo T, Wu F, Gao Y, Dong M, Liu M, Yang J, Li Y, Gong Q, Liang F, Kong J (2017). Acupuncture modulates the abnormal brainstem activity in migraine without aura patients. Neuroimage Clin.

[39] Wu Jiaping Gu, Shizhe.  (2011). Randomized controlled clinical observation of acupuncture in the treatment of migraine without aura. Acupuncture Res.

[40] Xiao L, Wang Y, Wang S, Wang L, Cui Q, Zhang C, Yao L, Shao L, Xing J. Clinical study of electroacupuncture in the treatment of migraine [J]. China Inf J Tradit Chin Med. 2018,25(01):19-22. (in Chinese). https://kns.cnki.net/kcms2/article/abstract?v=3uoqIhG8C44YLTlOAiTRKibYlV5Vjs7i0-kJR0HYBJ80QN9L51zrP2SvmETR-THzNyvsk4UJYZKoF9EngK6xg9e6EberO04J&uniplatform=NZKPT.

[41] Zhang Y, Wang Z, Du J, Liu J, Xu T, Wang X, Sun M, Wen Y, Li D, Liao H, Zhao Y, Zhao L (2021). Regulatory Effects of Acupuncture on Emotional Disorders in Patients With Menstrual Migraine Without Aura: A Resting-State fMRI Study. Front Neurosci.

[42] Zhao LP, Liu L, Pei P, Qu ZY, Zhu YP, Wang LP (2017). Electroacupuncture at Fengchi (GB20) inhibits calcitonin gene-related peptide expression in the trigeminovascular system of a rat model of migraine. Neural Regen Res.

[43] Junhui Z (2017). Observation on the application effect of acupuncture in the treatment of migraine. J Clin Rational Drug Use.

[44] Cook CL, Shedd GC (2018). Diagnosis and treatment of migraine in the patient with depression. J Am Assoc Nurse Pract.

[45] Silberstein SD (2004). Migraine. Lancet.

[46] Charles A (2018). The pathophysiology of migraine: implications for clinical management. Lancet Neurol.

[47] Burstein R, Noseda R, Borsook D (2015). Migraine: multiple processes, complex pathophysiology. J Neurosci.

[48] Noseda R, Jakubowski M, Kainz V, Borsook D, Burstein R (2011). Cortical projections of functionally identified thalamic trigeminovascular neurons: implications for migraine headache and its associated symptoms. J Neurosci.

[49] Goadsby PJ, Edvinsson L, Ekman R (1990). Vasoactive peptide release in the extracerebral circulation of humans during migraine headache. Ann Neurol.

[50] Yalınay Dikmen P, Onur Aysevener E, Kosak S, Ilgaz Aydınlar E, Sağduyu Kocaman A (2020). Relationship between MIDAS, depression, anxiety and alexithymia in migraine patients. Acta Neurol Belg.

[51] Ha H, Gonzalez A (2019). Migraine Headache Prophylaxis. Am Fam Physician.

[52] Silberstein SD (2015). Preventive Migraine Treatment. Continuum (Minneap Minn).

[53] Kelly RB, Willis J (2019). Acupuncture for Pain. Am Fam Physician.

[54] Jiang Y, Bai P, Chen H, Zhang XY, Tang XY, Chen HQ, Hu YY, Wang XL, Li XY, Li YP, Tian GH (2018). The Effect of Acupuncture on the Quality of Life in Patients With Migraine: A Systematic Review and Meta-Analysis. Front Pharmacol.

[55] Xu X, Liu L, Zhao L, Li B, Jing X, Qu Z, Zhu Y, Zhang Y, Li Z, Fisher M, Cairns BE, Wang L (2019). Effect of Electroacupuncture on Hyperalgesia and Vasoactive Neurotransmitters in a Rat Model of Conscious Recurrent Migraine. Evid Based Complement Alternat Med.

[56] Zhao L, Liu L, Xu X, Qu Z, Zhu Y, Li Z, Zhao J, Wang L, Jing X, Li B (2020). Electroacupuncture Inhibits Hyperalgesia by Alleviating Inflammatory Factors in a Rat Model of Migraine. J Pain Res.

[57] Cayir Y, Ozdemir G, Celik M, Aksoy H, Akturk Z, Laloglu E, Akcay F (2014). Acupuncture decreases matrix metalloproteinase-2 activity in patients with migraine. Acupunct Med.

[58] Tian Z, Guo Y, Yin T, Xiao Q, Ha G, Chen J, Wang S, Lan L, Zeng F (2021). Acupuncture Modulation Effect on Pain Processing Patterns in Patients With Migraine Without Aura. Front Neurosci.

[59] Vickers AJ, Vertosick EA, Lewith G, Macpherson H, Foster NE, Sherman KJ, Irnich D, Witt CM, Linde K (2018). Acupuncture for Chronic Pain: Update of an Individual Patient Data Meta-Analysis. J Pain.

[60] Wang C, Liu B, Liu Y, He L, Li H, Liu J (2018). [Analysis on the concepts related to adverse events and adverse reactions of acupuncture]. Zhongguo Zhen Jiu.

