# Supplementary material for: Effects of acupuncture on mental health of migraine patients: a systematic review and meta-analysis
Source: BMC Complement Med Ther. 2023 Aug 4;23:278. doi: 10.1186/s12906-023-04103-8 (PMC10401757; doi:10.1186/s12906-023-04103-8)
Supplement: Supplementary file 1 — Additional file 1. [file 12906_2023_4103_MOESM1_ESM.zip › Search strategy.pdf]

pubmed

#1 ((((((randomized controlled trial) OR (controlled clinical trial)) OR (randomized)) OR (placebo)) OR (clinical trials)) OR (randomly)) OR (trial)

#2 humans
#3 #1 AND #2
#4 ((headache disorders) OR (headache)) OR ((headache or migraine or cephalgia or cephalalgia or chronic migraine))

#5 (acupuncture therapy) OR ((acupuncture or body acupuncture or manual acupuncture or electroacupuncture or electro-acupuncture or auricular acupuncture or laser acupuncture or warm needling))
#6 #3 AND #4 AND #5

Web of science
#1 ((((((TS=(randomized controlled trial)) OR TS=(controlled clinical trial)) OR TS=(randomized)) OR TS=(placebo)) OR TS=(clinical trials)) OR TS=(randomly)) OR TS=(trial)

#2 TS=(humans)
#3 #1 AND #2

#4 ((TS=(headache disorders)) OR TS=(headache)) OR TS=(headache or migraine or cephalgia or cephalalgia or chronic migraine)

#5 TS=(acupuncture therapy)OR TS=(acupuncture or body acupuncture or manual acupuncture or electroacupuncture or electro-acupuncture or auricular acupuncture or laser acupuncture or warm needling)
#6 #3 AND #4 AND #5

Cochrane
Search Name:
Date Run: 21/12/2021 11:46:06
Comment:

ID	Search	Hits
#1	MeSH descriptor: [Randomized Controlled Trial] explode all trees	119
#2	MeSH descriptor: [Controlled Clinical Trial] explode all trees	128
#3	MeSH descriptor: [Random Allocation] explode all trees	20666
#4	MeSH descriptor: [Placebos] explode all trees	24507
#5	MeSH descriptor: [Clinical Trial] explode all trees	142
#6	randomly	277722

#7 trial 1370542
#8 #1 or #2 or #3 or #4 or #5 or #6 or #7 1409904
#9 humans 643867
#10 #8 and #9637604
#11 MeSH descriptor: [Headache Disorders] explode all trees 3606
#12 MeSH descriptor: [Headache] explode all trees 2513
#13 MeSH descriptor: [Migraine Disorders] explode all trees 2865
#14 cephalgia 49
#15 cephalalgia 1346
#16 chronic migraine 2069
#17 #11 or #12 or #13 or #14 or #15 or #16 7647
#18 MeSH descriptor: [Acupuncture Therapy] explode all trees 5100
#19 body acupuncture 1661
#20 manual acupuncture 844
#21 MeSH descriptor: [Electroacupuncture] explode all trees 860
#22 electro-acupuncture 703
#23 MeSH descriptor: [Acupuncture, Ear] explode all trees 209
#24 laser acupuncture 755
#25 warm needling 120
#26 #18 or #19 or #20 or #21 or #22 or #23 or #24 or #25 7581
#27 #10 and #17 and #26 190

Wan Fang

Subject =(migraine OR headache OR headache disease) AND subject =(acupuncture OR warm acupuncture OR acupuncture therapy OR body acupuncture OR dry acupuncture OR ear acupuncture OR manual acupuncture OR electroacupuncture OR electroacupuncture OR laser acupuncture) AND subject =(randomized controlled trial OR clinical effect OR randomized assignment OR clinical observation OR analysis OR impact)

CNKI

SU = (' migraine '+' headache '+' headaches) AND SU = (' acupuncture '+' warm needle '+' acupuncture '+' body acupuncture '+' dry needle '+' earpins' + 'manual acupuncture' + 'cupping' + 'cupping treatment' + 'laser needle) AND SU=(' Randomized controlled trial '+' clinical effect '+' randomized assignment '+' clinical observation '+' analysis '+' impact ')
